# The prevalence of oppositional defiant disorders among young people in europe: A systematic review and meta-analysis

**DOI:** 10.1192/j.eurpsy.2021.1698

**Published:** 2021-08-13

**Authors:** R. Sacco, N. Camilleri, K. Umla-Runge

**Affiliations:** 1 Psychiatry, Cardiff University, Teesside University, Malta Mental Health Services, Attard, Malta; 2 Child And Young People’s Services, Malta Mental Health Services, Pieta, Malta; 3 Psychiatry, Cardiff University, Cardiff, United Kingdom

**Keywords:** Child, prevalence, Europe, oppositional defiant disorder

## Abstract

**Introduction:**

This systematic review estimates the pooled prevalence (PP) of oppositional defiant disorders (ODD) among 5-to-18-year-old YP living in Europe, based on prevalence rates established in the last five years (LFY).

**Objectives:**

Trends of prevalence rates across countries, gender and level of education were analysed. The random effects pooled prevalence rate (REPPR) for ODD was calculated.

**Methods:**

A search strategy was conducted on three databases. Studies were also identified from reference lists and grey literature. Eligible studies were evaluated for reliability, validity, bias, and the REPPR for ODD was calculated.

**Results:**

The European REPPR for ODD is calculated at 1.9% (Figure 1). The REPPR among males is 4.8%, whereas the rate among females is 2.7% (95% CI: 0.7%- 1.4%). The prevalence rate of ODD among primary school children is 1.8 times higher than the prevalence of secondary school children (Figure 2).

**Figure:**
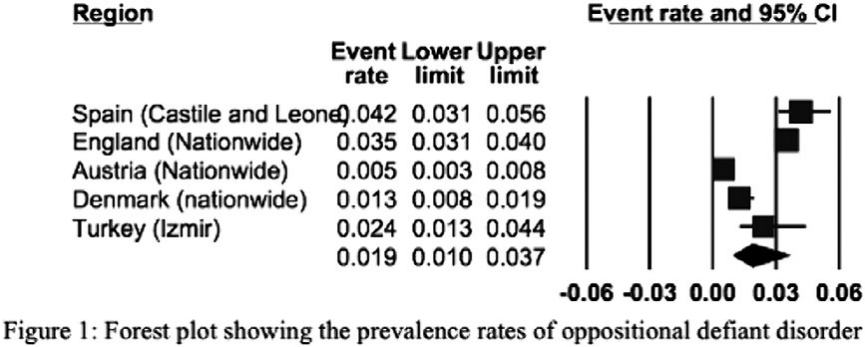

**Figure:**
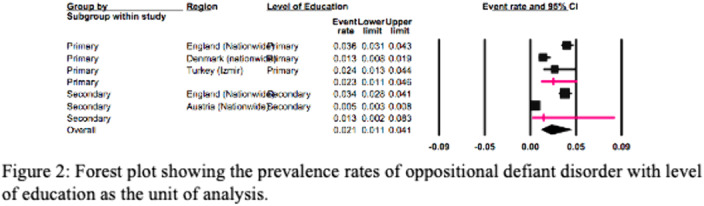

**Conclusions:**

Gender, culture and socioeconomic diagnostic inequality may contribute to prevalence differences across countries. Routine screening and addressing these aspects may facilitate early intervention.

**Disclosure:**

No significant relationships.

